# Characteristics and mortality of patients categorised with non-specific symptoms when dialling the emergency medical number: a register-based cohort study

**DOI:** 10.1186/s12873-025-01311-y

**Published:** 2025-08-15

**Authors:** Vilde Fosso Smievoll, Helene Monsen Folkedal, Lars Myrmel, Guttorm Brattebø

**Affiliations:** 1 Faculty of Medicine, University of Bergen, Bergen, Norway; 2 Department of Anaesthesia and Intensive Care, Bergen Emergency Medical Services, Haukeland University Hospital, Bergen, Norway; 3 Department of Anaesthesia and Intensive Care, Haukeland University Hospital Bergen, Bergen, Norway; 4 Norwegian National Advisory Unit on Emergency Medical Communication, Haukeland University Hospital, Bergen, Norway

**Keywords:** Non-specific problem, Non-specific symptoms, Prehospital emergency medicine, Emergency medical communication centre, Health services

## Abstract

**Background:**

Medical communication centre operators of the 1-1-3 medical emergency number in Norway use the decision support tool, the Norwegian Index for Medical Emergency, to categorise the problem and determine the correct handling and urgency level of the situation. The index comprises 42 chapters, one of which is titled ‘Non-specific problem’. Studies in Denmark frequently use this chapter; however, there are no published Norwegian studies on the demographics of this patient group. Thus, we investigated the characteristics of the patients assigned to this chapter and their 1- and 30-day mortality outcomes.

**Methods:**

This was a registry-based, retrospective cohort study. Descriptive statistics were used to compare the two groups; t-tests were performed for continuous variables, and data were presented with corresponding 95% confidence intervals. Categorical data were compared using the chi-square test. Statistical significance was set at *p* < 0.05.

**Results:**

Out of the 25,474 included calls to the emergency medical communication centre in 2022, 1,860 (7.3%) were categorised as ‘Non-specific problem’. Patients in this group had a higher mean age, were more often men, had a shorter hospital stay, and showed more comorbidities than the control group. The use of this chapter was associated with the allocation of a higher urgency level, and a reduction in the use of these criteria was observed during nighttime. Significantly higher 1- and 30-day mortality rates were observed in patients with non-specific symptoms (1.40% and 6.94%, respectively; *p* < 0.05).

**Conclusions:**

The group presenting symptoms categorised as non-specific comprised older patients, more men, and a higher number of patients showing comorbidities than the control group. Patients presenting symptoms categorised as non-specific typically perceived a high level of urgency. Most of these patients had a non-specific main diagnosis after hospital admission and a significantly higher mortality rate than those presenting with symptoms categorised as specific.

**Clinical trial number:**

Not applicable.

**Supplementary Information:**

The online version contains supplementary material available at 10.1186/s12873-025-01311-y.

## Background

Early diagnosis, correct treatment, and fast transportation to the right level of healthcare are the foundations of prehospital treatment. These aspects increase the probability of a good outcome for the patient [1–3]. Operators in emergency medical communication centres (EMCC) serve an important role in ensuring that correct care is provided timely [4]. EMCC operators use a criterion-based decision support tool called the Norwegian Index for Medical Emergency (Index) to define the health problems of patients who call in [5–8]. This tool identifies the medical problem, aids the operator in determining the correct response, and indicates the level of urgency of the situation [5, 8].

Most of the listed chapters in the Index are symptom-specific, except for the chapter listed as number 07: ‘Non-specific problem’ (NSP) [8]. Danish studies have shown that patients allocated to this Index chapter have higher age and higher mortality compared to other Index chapters, as well as one study indicating overuse of the NSP chapter [9–11]. However, there are no published Norwegian studies on the demographics of this patient group. Owing to challenges related to defining the problem among patients in the NSP group, this group may be at risk of delayed diagnosis; therefore, correct treatment initiation may be prolonged. Patients in the NSP group are older and have a higher mortality rate than those in the control group presenting with specific problems [9–12]. A higher incidence of over- and under-triage has been reported among patients in the NSP group than in the control group [13].

Based on this knowledge, we investigated patients allocated to the NSP criteria in our Emergency Medical System (EMS). We aimed to compare patient characteristics, mortality and length of hospital stay between the NSP group and patients presenting symptoms categorised as specific.

## Methods

### Study design and data sources

We conducted a register-based retrospective cohort study to describe a patient group with symptoms categorised as NSP when calling the EMCC. Throughout this article, patients presenting symptoms categorised as NSP by the EMCC personnel will be referred to as the NSP group. The data included routinely collected patient information from all medical emergency calls made to the Bergen EMCC in Norway over 1 year (2022). Data were collected from two different registers: the EMCCs patient records (AMIS) and the hospital’s electronic patient record (EPJ) system (DIPS).

### Setting

Helse Bergen HT is located in the western part of Southern Norway and provides specialised healthcare, including EMCC and ambulance services, to approximately 470,000 inhabitants [5, 14]. The trust covers a total land area of approximately 10,000 km^2^, including cities, islands, fjords, and mountains. The 18 municipalities comprising the trust have a population density varying from 1 to 650 inhabitants per km^2^ [14, 15]. The region has three hospitals; only one has a trauma centre and a PCI centre [16, 17].

A person in need of pre-hospital medical assistance in Norway may contact the nearest EMCC by dialling the free-of-charge medical emergency number, 1-1-3. The patient contact may also be directed to the EMCC from a general practitioner or Emergency Primary Care Centre. The EMCCs are staffed by specially trained ambulance personnel and nurses. When receiving a call, the EMCC operator uses the index to identify the medical problems [5, 7]. The Index is a criteria-based decision support tool comprising 42 chapters, with each chapter containing a specified list of signs, symptoms, and situational descriptions to guide the operator in determining the correct level of urgency, and dispatching the appropriate resources needed for the situation at hand [5, 8]. The urgency levels are categorized as acute (red), urgent (yellow), and non-urgent (green). Acute (red) indicates a time-critical and potentially life-threatening situation in need of immediate medical assistance. Urgent (yellow) refers to a non-life-threatening situation but in need of medical assistance without delay. Non-urgent (green) encompasses scheduled patient transport.

### Participants

**Fig. 1:**
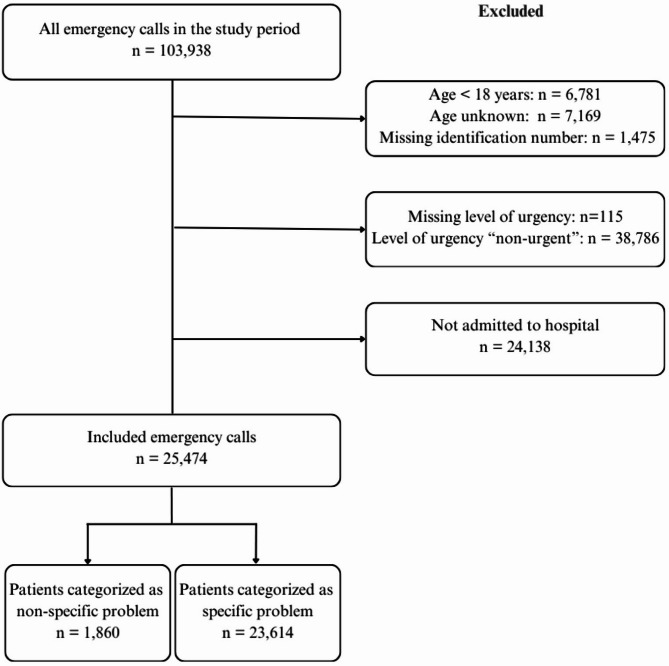
Medical emergency calls included and excluded in the study

Patients aged under 18 years, those with unknown age or ID, and those with missing information on the level of urgency, and patients with urgency level ‘non-urgent’, were excluded (Fig. 1). Patients who received prehospital treatment but were not admitted to the hospital, and therefore did not receive any ICD-10 diagnoses, were also excluded. The remaining calls were subsequently split into two groups: emergency calls categorised as NSP according to the Index, and a control group containing all emergency calls other than ‘non-specific problem’.

### Variables and outcomes

Patient data on age, sex, length of hospital stay, diagnosis at discharge, and number of days until death were extracted from the EPJ. Information on the urgency level, time of contact, criterion number, and delivery location were collected from the EMCC’s digital system. Time of contact refers to the initial contact with EMCC. Length of stay was calculated from the date and time of admission (dd: mm: yyyy hh: mm) to the time of discharge. These data were regenerated from the hospital electronic patient record system. Time of death was collected from the Cause of Death Register and represents the duration from hospital admission to death.

Differences in the occurrence of calls throughout the day were analysed by comparing the total number of calls during the day, evening, and night; the time intervals 07.00 am to 02.59 pm, 03.00 pm to 09.59 pm and 10.00 pm to 06.59 am were used, respectively, as they represent the typical hours of a three-part rotation often used in the Norwegian health care system [18]. We investigated the number of calls every weekday, compared between weekdays (Monday to Friday) and weekends (Saturday to Sunday), and the monthly variation in calls throughout the year.

Mortality rates were calculated as deaths within 1 and 30 days of the call. As Norwegian hospitals use the World Health Organization (WHO) International Classification of Diseases 10th Revision (ICD-10) when discharging patients, we used the main chapters of ICD-10 in our analysis of diagnoses [19, 20]. The first listed diagnosis was used as the patient’s main diagnosis, and the total number of diagnoses at discharge was used to measure comorbidity, assessing those with 1, 2–3, or 4 or more diagnoses.

### Statistical analysis

The data extracted from the two registers were aligned using national individual identification numbers, de-identified, and stored on a secure hospital server. The data were stratified into two groups as shown in Fig. 1, and descriptive statistics were used for between-group comparisons. Analysis of age and length of hospital stay included range, mean, and interquartile range, and the proportions of males and females in the groups were determined. T-tests were performed for continuous variables, and data were presented with corresponding 95% confidence intervals (95% CIs). Categorical data were compared using the chi-square test. Statistical significance was set at *p* < 0.05.

### Ethics

The need of approval for this study was waived by the regional research ethics committee because it was viewed as quality improvement (2023/606320/REK vest). Hence, according to national regulations it required acceptance from the involved hospital department and the hospital data protection officer, which was secured. The study was conducted in accordance with the declaration of Helsinki.

## Results

In 2022, the Bergen EMCC received 103,938 emergency calls, of which, 25,747 calls met our inclusion criteria (Fig. 1). The included calls were divided into two groups: NSP (7.3%) and control with symptoms categorised as specific by the EMCC (92.7%).

Patient characteristics and dispatch information for the two groups are shown in Table 1. Notably, a significantly higher mean age was observed in the NSP group (66.0 years) than in the control group (61.7 years) and the proportion of men was significantly higher in the NSP group. A shorter length of hospital stay was observed in the NSP group (3.49 days) than in the group with specific problems (3.99 days). A higher level of comorbidity was found among patients in the NSP group, where most received four or more diagnoses at discharge (38.1%), compared with the control group, with most patients receiving two to three diagnoses (39.0%).

**Table 1:** Variables describing patients contacting the medical emergency number, categorised with a non-specific or specific problem

Variables		Non-specific	Specific	*P*-value
		*N* = 1860	*N* = 23,614	
*Age in years*	*Range (IQR)*	18–103 (53–82)	18–108 (44–80)	
	*Mean (95% CI)*	66.0 (65.11–66.98)	61.7 (61.38–61.94)	< 0.05*
*Sex*	*Female*	841 (45.2%)	11,283 (47.8%)	< 0.05*
	*Male*	1019 (54.8%)	12,331 (52.2%)	
*Length of stay*	*Range (IQR)*	0–150 (1–4)	0–388 (0–4)	
	*Mean (95% CI)*	3.49 (3.17–3.80)	3.99 (3.885–4.12)	< 0.05*
*Comorbidity*	*1 diagnose*	451 (24.3%)	7146 (30.3%)	< 0.05*
	*2–3 diagnoses*	701 (37.7%)	9219 (39.0%)	
	*4 + diagnoses*	708 (38.1%)	7249 (30.7%)	
*Time of day*	*Daytime*	853 (45.9%)	9677 (41.0%)	< 0.05*
	*Evening*	671 (36.1%)	8345 (35.3%)	
	*Night*	336 (18.1%)	5592 (23.7%)	
*Day of the week*	*Monday*	248 (13.3%)	3404 (14.4%)	0.38
	*Tuesday*	292 (15.7%)	3336 (14.1%)	
	*Wednesday*	269 (14.5%)	3296 (14.0%)	
	*Thursday*	240 (12.9%)	3287 (13.9%)	
	*Friday*	263 (14.1%)	3346 (14.2%)	
	*Saturday*	269 (14.5%)	3509 (14.9%)	
	*Sunday*	279 (15.0%)	3436 (14.6%)	
	*Weekdays*	1312 (70.5%)	16,669 (70.6%)	0.96
	*Weekend*	548 (29.5%)	6945 (29.4%)	
*Month*	*January*	142 (7.63%)	1821 (7.71%)	0.13
	*February*	139 (7.47%)	1733 (7.34%)	
	*March*	153 (8.23%)	1886 (7.99%)	
	*April*	138 (7.42%)	1897 (8.03%)	
	*May*	185 (9.95%)	1988 (8.42%)	
	*June*	163 (8.76%)	2003 (8.48%)	
	*July*	133 (7.15%)	2050 (8.68%)	
	*August*	183 (9.84%)	2021 (8.56%)	
	*September*	149 (8.01%)	2132 (9.03%)	
	*October*	161 (8.66%)	2144 (9.08%)	
	*November*	162 (8.71%)	1984 (8.40%)	
	*December*	152 (8.17%)	1955 (8.28%)	
*Urgency level*	*Acute*	968 (52.0%)	10,156 (43.0%)	< 0.05*
	*Urgent*	892 (48.0%)	13,458 (57.0%)	
*1-day mortality*	*Acute*	21 (2.17%)	157 (1.55%)	0.14
	*Urgent*	5 (0.56%)	47 (0.35%)	0.31
	*Total*	26 (1.40%)	204 (0.86%)	< 0.05*
*30-day mortality*	*Acute*	80 (8.26%)	585 (5.76%)	< 0.05*
	*Urgent*	49 (5.49%)	552 (4.10%)	< 0.05*
	*Total*	129 (6.94%)	1137 (4.81%)	< 0.05*

*Statistically significant, *p* < 0.05

A significantly larger proportion of calls in the NSP group were triaged as ‘acute’, compared with the calls categorised with a specific problem – at 52% and 43%, respectively. Reasons for assigning urgency levels in the NSP group are provided in supplemental file 1. Analysis of the distribution of calls throughout the day revealed a lower occurrence of the chapter ‘non-specific problem’ during nighttime, compared with the symptom-specific chapters. However, no significant differences were found in the occurrence of calls throughout the week when comparing weekdays with weekends, and there was no significant variation in the monthly distribution of calls throughout the year.

Furthermore, significantly enhanced mortality was observed in the NSP group than in the control group after 1 and 30 days, (Table 1). There was no significant difference in 1-day mortality rates between the ‘acute’ and ‘urgent’ urgency levels. However, significantly increased 30-day mortality was observed in the NSP group at both urgency levels. The largest differences in mortality were found among patients triaged with the urgency level ‘acute’, with 30-day mortality being 8.26% in the NSP group and 5.76% in the control group.

### Diagnosis at discharge from hospital

All 21 ICD-10 chapters were used as main diagnoses at discharge from the hospital in both groups, except Chap. 16, ‘Certain conditions originating in the perinatal period’, and Chap. 20 ‘External causes of morbidity’. As shown in Table 2, Chap. 18 ‘Symptoms, signs, and abnormal clinical and laboratory findings, not elsewhere classified’ was the most frequently used for patients in the NSP group, followed by Chaps. 9 ‘Diseases of the circulatory system’, 19 ‘Injury, poison and certain other consequences of external causes’, 10 ‘Diseases of the respiratory system’, and 5 ‘Mental, behavioural and neurodevelopmental disorders’. The same five chapters were most frequently used in the control group, albeit in a different order.

**Table 2:** Top-five ICD-10 chapters at hospital discharge and their distribution among patients categorised as non-specific/specific problems

Chapter	Title	Non-specific (*N* = 1860)	Specific (*N* = 23,614)
5	Mental, behavioural and neurodevelopmental disorders	9.8%	12.4%
9	Diseases of the circulatory system	14.2%	13.1%
10	Diseases of the respiratory system	10.7%	10.7%
18	Symptoms, signs, and abnormal clinical and laboratory findings, not elsewhere classified	17.3%	12.8%
19	Injury, poison and certain other consequences of external causes	12.5%	21.7%

## Discussion

### Characteristics of patients categorised with a non-specific problem

This study aimed to describe the demography of patients with NSP and investigate their outcomes measured based on 1- and 30-day mortality rates. We found that the NSP group had a statistically significant higher mean age, comprised more men, showed a shorter length of hospital stay, and had higher comorbidities than did patients with a specific problem. Although the differences were statistically significant, the clinical significance of sex, length of stay and 1-day mortality should not be overstated. The NSP group was triaged to a higher urgency level, and fewer calls were categorised as NSP at night, compared with the control group. No significant differences were found in the distribution of calls throughout the week, between weekdays and weekends, or across months. However, higher 1- and 30-day mortality rates were observed in the NSP than the control group.

A total of 7.3% of the calls made to the EMCC in 2022 were categorised as NSP – lower than similar reports from Denmark, where the proportions ranged from 8 to 18% [9–11]. Our finding of higher mean age in the NSP group correlated well with those of previous studies [9–11]. We found significantly higher comorbidity in the NSP group. Increased comorbidities could be a complicating factor in the medical assessment of patients. It could also be assumed that older adult patients have higher frailty and are thus at a higher risk of morbidity and mortality. As reported in a study of older adult patients visiting the emergency room by Wachelder et al., several factors, such as comorbidities, communication problems, and cognitive impairment, affect the compilation of history, making assessing and triaging patients more difficult [21].

Patients in the NSP group were triaged to a higher level of urgency than were those in the control group (Table 1).

Our findings of a higher level of triage and a higher mortality rate in the NSP group may indicate that these patients could present with more severe disease than the control group. This could also reflect that patients with diffuse or uncertain symptoms are over-triaged.

### Mortality

Our study revealed significantly increased mortality among patients in the NSP group than among those in the control group, both in 1- (1.40%) and 30-day mortality (6.94%) rates (Table 1). Stratification of the number of deaths within the two urgency levels revealed that only urgency level and 30-day mortality were associated. Within the NSP group, a significantly increased 30-day mortality was observed among patients triaged as ‘acute’ and ‘urgent’. A higher mortality rate in the NSP group could be expected due to differences in age and comorbidity.

The increased mortality observed in the NSP group correlates well with findings in other studies, where 1-day mortality was reported to be between 2.2 and 2.3%, and 30-day mortality ranged from 6.0 to 7.1% [10, 12]. A study from the Netherlands on older adult patients admitted and categorised with non-specific problems found an increased 30-day mortality rate compared with patients categorised with specific complaints [21]. Møller et al. analysed mortality rates across urgency levels and found an association between lower priority level (B-calls) and increased mortality for patients with an unclear problem as compared to patients with a specific problem [11].

### Diagnoses at discharge

When analysing the patients’ main diagnosis at hospital discharge, we found the same five ICD-10 chapters to be the most frequently used, both in the NSP and control groups, although in different orders. Four of these chapters represent specific diagnoses, whereas Chap. 18 ‘Symptoms, signs and abnormal clinical and laboratory findings, not elsewhere classified’, lists more abnormal symptoms not associated with a specified diagnosis [19]. This chapter was most frequently used among patients categorised as having an NSP (17.1%). In contrast, it was only the third most used chapter among patients categorised as having a specific problem (12.8%).

Our findings also suggest that almost one out of five patients with an NSP prehospitalisation were discharged from the hospital with a non-specific diagnosis. Patients with an unclear problem after a thorough examination at the hospital might understandably constitute a difficult patient group to categorise prehospitalisation. The rest of the group received more specific diagnoses when examined at the hospital, and these patients could potentially be better categorised at first contact. The difficulties in categorising the problem of this patient group may be related to communication problems, weaknesses in the decision support tool used by the EMCC, or an atypical presentation of the symptoms or disease at the time of contacting the EMCC. However, further research is required to confirm these reasons.

### Strengths and limitations

Every Norwegian has a personal identification number, aiding in reliably linking data across different record systems. As a result of the digitalisation of the prehospital journal system in 2021, the linkage between registries is more reliable than the prehospital paper-based patient records utilised previously by the EMS. The improved linkage between prehospital and hospital records strengthens accuracy and lowers information and selection bias. As the study was based on register-based data, information and selection biases were low.

Our study has some limitations. As this was a retrospective registry-based study, it was susceptible to known and unknown confounding factors. Despite reliable records, several patients had missing data and, consequently, had to be excluded (*n* = 16639, 16%). These exclusions may have confounded the results [22]. However, when reviewing the patient data, a frequent explanation listed in the registry column describing the caller’s presenting problem was ‘Misdialled’ or ‘No answer’, which may explain a large number of calls being excluded. As Norway has a universal healthcare system, the findings may be limited to countries with similar healthcare systems, thus reducing generalisability of the results. However, the universal healthcare system reduces the risk of selection bias.

Furthermore, there were limitations to the analysis of diagnoses at discharge. As hospitals in Norway are financially compensated at a fixed rate tied to the various discharge diagnoses, it is possible that the first diagnosis is not the main diagnosis but the one triggering the largest economic compensation. However, this cannot be confirmed without reviewing the patients’ hospital records. Finally, the number of discharge diagnoses was used to indicate comorbidity within the two groups, which may be a poor variable for comorbidities at the individual level. However, it may indicate comorbidities at the group level. The use of a validated comorbidity index, ex. Charlson comorbidity score, which has been used in the Danish studies, would require access to the individual patient records. Due to ethical approval, we had no access to the individual patient hospital records.

## Conclusions

In summary, 7.3% of the patients who contacted the EMS because of a medical emergency in 2022 were categorised according to the NSP prehospital criteria. This group comprised older patients, more men, and more patients with comorbidities. They typically had a higher level of urgency than the control group categorised by any criteria other than NSP. Most of these patients received a non-specific main diagnosis after hospital admission with significantly higher 1- and 30-day mortality rates than patients with complaints categorised as specific. Our findings indicate that the current decision support tool may need to be modified to better classify the callers’ conditions, and to better suit the EMCC operators’ approach to it.

## Supplementary Information

Below is the link to the electronic supplementary material.

Supplementary Material 1

## Data Availability

Data are available from the corresponding author on reasonable request.

## Abbreviations

95% CI: 95% confidence interval
EMCC: Emergency medical communication centre
EMS: Emergency medical system
EPJ: Electronic patient record
ICD-10: International Classification of Diseases 10th Revision
Index: Norwegian Index for Medical Emergency
IQR: Interquartile range
NSP: Non-specific problem
PCI: Percutaneous coronary intervention
REK: Regional Committees for Medical and Health Research Ethics
WHO: World Health Organization
