# Supplementary material for: Characteristics and mortality of patients categorised with non-specific symptoms when dialling the emergency medical number: a register-based cohort study
Source: BMC Emerg Med. 2025 Aug 15;25:156. doi: 10.1186/s12873-025-01311-y (PMC12357406; doi:10.1186/s12873-025-01311-y)
Supplement: Supplementary file 1 — Supplementary Material 1 [file 12873_2025_1311_MOESM1_ESM.docx]

| Criteria | Chapter name | Number of calls |
| --- | --- | --- |
| A.07.01 | Does not respond to shaking and shouting | 105 |
| A.07.02 | Breathing problems | 26 |
| A.07.03 | Still conscious, but sudden faintness / dizziness | 79 |
| A.07.04 | Pale and clammy skin, sudden onset | 48 |
| A.07.05 | Significantly ill or weak, sudden onset | 276 |
| A.07.06 | Unwell and suddenly very frail | 27 |
| A.07.07 | Assume serious / critical problem (more information not readily available) | 249 |
| A.07.08 | Difficulty communicating, assume serious / critical problem | 66 |
| H.07.01 | Exhausted patient (uncertain / unclear information) | 262 |
| H.07.02 | Intense pain | 106 |
| H.07.03 | Fainted several times, better now | 35 |
| H.07.04 | Assistance required via home alarm system | 6 |
| H.07.05 | Functional impairment / rapid decline, recent onset | 211 |
| H.07.06 | Assume urgent problem (more information not readily available) | 135 |
| H.07.07 | Assume urgent problem (lacking anything applicable in NINM) | 172 |
| H.07.08 | Repeated calls, unclear problem | 7 |
| H.07.09 | Difficulty communicating, unclear problem | 40 |
|  | Total | 1850 |
